# Biomarkers of traumatic brain injury: narrative review and future prospects in neurointensive care

**DOI:** 10.3389/fmed.2025.1539159

**Published:** 2025-06-03

**Authors:** Marta Pryzmont, Urszula Kosciuczuk, Mateusz Maciejczyk

**Affiliations:** ^1^Department of Anaesthesiology and Intensive Therapy, Medical University of Bialystok, Białystok, Poland; ^2^Department of Hygiene, Epidemiology, and Ergonomics, Medical University of Bialystok, Białystok, Poland

**Keywords:** brain, critical care, exosome, neuroprognosis, neurotrauma

## Abstract

Traumatic brain injury (TBI) is a significant medical problem because of its high early mortality rate in intensive care and high risk of severe neurological complications in long-term follow-ups. Craniocerebral injuries are one of the most important issues in intensive therapy due to the limited prognostic possibilities for the neurological consequences of such injuries. Computed tomography and magnetic resonance imaging are the most common and available radiological tools for presenting and describing morphological brain damage in the acute and chronic phases of TBI. The use of biomarkers may improve the accuracy of establishing the severity and prognoses in patients with severe traumatic brain damage. Based on the available publications, there is no definitive and accurate single marker that has high prognostic value regarding neurological brain tissue damage; however, the combination of several biomolecules (i.e., biomarkers of neuronal, astrocyte, and cytoskeleton disruption and chemokines) significantly increases the diagnostic value. Most scientific studies are based on serum and cerebrospinal fluid assays. This publication presents the current state of the knowledge about the markers of nervous tissue damage in the brain and their clinical utility in mortality prediction and neurological prognosis in critical neurointensive care. Moreover, this review article presents the correlations between the biomarkers, radiological signs of brain injury, and clinical scales, as well as the latest scientific and publication trends, such as microRNA genetic studies and different laboratory assay methodologies using various biological materials.

## 1 Introduction

According to the World Health Organization, traumatic brain injury (TBI) is one of the most common causes of death and disability in the world, with more than 10 million hospitalizations annually (1, 2). Epidemiological data from the United States indicate 1.4 million TBI cases per year (3). For European populations, the TBI incidence is 235 per 100,000 per year, with a mortality rate of 0.015% (4). Traumatic brain injury is particularly common among people below 25 and above 75 years of age (5, 6), and in most of the reviewed studies, it is more prevalent among men than women (7, 8). The most common causes of traumatic brain injury in European populations include falls, mostly in children and elderly people, and road accidents, which are the leading cause of TBI among young adults. Additional causes include battery and sports- and recreation-related injuries (4).

TBI is a significant medical problem because it is characterized by high short-term mortality and a high risk of severe neurological complications in long-term follow-ups, and there is the risk of chronic cognitive and behavioral disorders, consciousness disorders, chronic traumatic encephalopathy, physical disability, psychiatric and neurodegenerative diseases, and organ dysfunction secondary to neurological deficits (9–12).

The assessment methods for brain injury are complex and presented as classification scales, such as the Abbreviated Injury Scale (AIS), Injury Severity Scale (ISS), Revised Trauma Score (RTS), or local injury assessments (the Mayo Classification System for TBI), and they are based on general neurological examinations and imaging scans (13, 14).

The Mayo Classification System for TBI is a typical TBI classification system. Possible TBI is connected with neurocognitive symptoms, such as blurred vision, confusion, headache, nausea, and a loss of consciousness for <30 min. According to the Mayo Classification System for TBI, a TBI is classified as mild (probable) if one or more of the following criteria apply: the loss of momentary consciousness to <30 min, the post-traumatic anterograde amnesia of momentary consciousness to <24 h, depression, and basilar or linear skull fracture (with the dura intact). The most serious TBI cases are moderate and severe TBI, according to the Mayo Classification System for TBI, and they are recognized when one or more of the following criteria apply: death due to TBI, loss of consciousness for 30 min or more, post-traumatic anterograde amnesia for 24 h or more, a worsening Glasgow Coma Scale full score of <13 in the first 24 h [unless invalidated upon review (e.g., attributable to intoxication, sedation, systemic shock)], and one or more of the following present: an intracerebral hematoma, a subdural hematoma, an epidural hematoma, a cerebral contusion, a hemorrhagic contusion, dura penetration of the TBI, subarachnoid hemorrhage, and brain stem injury (15–17).

The most common neurological scales used for neurological examinations include the Glasgow Coma Scale (GCS) and the Glasgow Coma Scale—Pupils (GCS-P). The Glasgow Coma Scale (GCS) is used to assess the extent of the best motor response, verbal response, and eye opening, and it allows for tentative determinations of TBI prognoses. Depending on the GCS score, the course of traumatic brain injury can be classified as mild (a GCS score of 13–15), moderate (a GSC score of 9–12), or severe (a GCS score of 3–8). The long-term neurological state is determined using the Glasgow Outcome Scale (GOS) and Glasgow Outcome Scale—Extended (GOSE) (15–17).

Primary brain injuries are classified with the qualitative Marshall scale and the Rotterdam CT score. The Marshall scale involves six categories of brain injury depending on computed tomography (CT) images: Category 1 includes cases wherein there are no intracranial pathologies in the CT image; Category 2 includes cases that have a midline shift of <5 mm; Category 3 describes cases of compressed or effaced basal cisterns with a midline shift of <5 mm; Category 4 indicates diffuse injuries that involve a midline shift of >5 mm; and Categories 5 and 6 pertain to cases of surgical mass evacuation, where Category 5 applies to every surgically treated injury, and Category 6 describes injuries of high or mixed density (over 25 mL) that have not been surgically treated (17, 18). The Rotterdam CT score is a classification that has been described relatively recently. The scale is used to assess CT images, pathological lesions, blood in the ventricular system, and subarachnoid bleeding using categories ranging from 0 to 6. Categories 5 and 6 include unfavorable prognoses for patients with TBI (19, 20).

Radiological imaging tests are crucial for determining the nature of brain injuries, their locations, and the indications for surgical treatment. However, many publications have stated that repeated CT scans of the brain are unnecessary in approximately 35% of cases, especially among patients without deterioration in their neurological condition and in cases of mild brain injuries and axonal damage. Imaging data are only one element of urgent diagnostics; thus, they are not sufficient for understanding the mechanism of the injury and cannot serve as the basis for long-term prognoses. Furthermore, the analysis of biological material in patients with TBI in Intensive Care Units (ICUs) has recently garnered significant interest, as it reduces the risks typically associated with transporting intensive care patients to radiology departments. However, biomarkers offer a more comprehensive approach via the analysis of the levels of specific biomarkers to gain a more complete understanding of the disruption of the neural structure integrity, the regenerative capacity of the neural tissue, and the reconstruction and myelination of nerve fibers, neurons, and astrocytes. Therefore, the evaluation of TBI biomarkers complements radiological imaging techniques with the assessment of possible future neurological deficits, thereby allowing for the appropriate course of therapy to be determined and the selection of the optimal, patient-specific rehabilitation, offering the chance to achieve better results (21).

Brain injury biomarker determination is an important supplement to classification and imaging methods. Although there are qualitative methods for assessing brain injuries, the determination of the biomarker concentration is an example of a quantitative method. The performance of biomarker diagnostics has been suggested as a method for quantitatively approaching the issue of prognosis and directly determining the pathophysiological mechanisms of the injury (2). The most frequently used biological materials are serum and cerebrospinal fluid (CSF). Both methods are characterized by invasive entry and the risk of infection. The concentrations of biomarkers are used to diagnose the acute and long-term complications of brain injuries and assess their cellular and molecular mechanisms, thereby improving prognoses and clinical assessments. Additionally, the repeatability of the tests and the short time required to complete laboratory determinations add to the usefulness of these methods (21, 22).

Studies over the last two decades indicate a significantly increased interest in the topic of biomarkers in TBI, with the most common publications presenting nerve tissue protein, cytokine, and coagulation tests. Moreover, TBI biomarkers in different biofluids have also been discovered (10, 23–25).

## 2 Objectives and methods

The aims of this review were to analyze the current state of the knowledge and describe the available biomarkers of traumatic brain injury and their correlation with the stages of brain injury and the clinical prognosis. The literature search was conducted up to 1 July 2024. The PubMed and Cochrane databases were used to identify studies published in English that focused on TBI epidemiology and pathophysiology and the biochemical analysis of TBI biomarkers. The literature search revealed 351 articles from the PubMed database and 41 articles from the Cochrane database. Only international publications written in English were selected. A total of 271 items were excluded, including articles and abstracts. This review included selected literature reviews, as well as observational, experimental, and clinical studies published between 2010 and 2024.

## 3 Central nervous system markers

Brain tissue is a complex collection of neuronal cells and accessory elements that are isolated via the blood–brain barrier (BBB) (26). The pathophysiology of traumatic brain injury results from a disruption in the integrity of neural structures [i.e., neuronal bodies and nerve fibers (axons)], as well as from disruption in the cytoskeleton (microtubules and microfilaments) and structure-providing elements (27–29). Mechanical injury causes biological (disintegrative) injury, which then leads to dynamic biochemical changes. Blood–brain barrier leakage results in the secretion of stored and newly synthesized mediators into systemic circulation, allowing for the determination of brain tissue injury mediators in sera. Local biochemical mediators are augmented by systemic mediators and assume the form of the multidirectional and dynamic effects of oxidative stress, oxygen free radicals, interleukins, and apoptotic factors (30–32). The following mediators have been specified: mediators related to the biochemical injury of astroglial cells, neuronal damage demyelination processes, and axonal injury neurodegenerative processes and cytokines (31–37).

### 3.1 Biochemical markers of astroglial cell damage

S100B is the most well-known and best-described protein that is used as an astroglial cell injury marker. S100B is a type of calcium-binding protein that is composed of two chains (the alpha and dominant beta chains), with a molecular weight of 21 kDa, and it is mostly expressed in astrocytes (38); however, it is peripherally found in lower concentrations in adipocytes, chondrocytes, and monocytes. Under normal conditions, the beta molecule minimally permeates the blood–brain barrier, with a CSF/serum ratio of 18:1; however, if traumatic brain injury occurs, then the molecule is released from the damaged glial cells and diffuses into the bloodstream through the leaking BBB. In addition, its serum half-life is about 30–90 min, which increases (i.e., up to 24 h) in cases of severe TBI (39). The S100B protein interacts with the receptor for advanced glycation end products (RAGE). In the extracellular environment, the protein has a protective and neurotrophic effect, stimulating nerve fiber overgrowth and promoting neuronal vitality (40). Bianchi et al. (41) report the significant role of the S100B protein in astrocyte–neuron communication, showing the neuroprotective action of the protein at the initial stage of brain injury. Under normal conditions, serum S100B protein levels range from 0.06 to 0.13 μg/L, and values from 0.07 to 0.24 μg/L indicate astrocyte damage that is secondary to TBI (42). Additionally, concentrations above 0.16 μg/L are characterized by the best specificity in terms of predicting the radiological changes in CT images of mild TBI. It has also been reported that S100B concentrations are correlated with unfavorable survival prognoses and the overall poor neurological prognoses of severe TBI (21).

The S100B protein is eliminated via the renal function, and its serum levels remain stable for up to 8 h at room temperature and for up to 48 h at 2–8°C, which makes it an attractive molecule as a clinical TBI biomarker for analysis (43, 44). Moreover, S100B is a reliable biomarker, as it is relatively unaffected by external environment conditions, hemolysis, or storage conditions. The S100B serum concentration is stable for up to 8 h at room temperature and for 48 h between 2 and 8°C. The two main laboratory methods are the enzyme-linked immunosorbent assay (ELISA) and electrochemiluminescence (ECL) assay (45). S100B analyses are recommended as part of the Scandinavian and French guidelines for the Initial Management of Minimal, Mild, and Moderate Head Injuries in Adults. An S100B level that is <0.1 ug/L rules out the need for brain CT scans within 6 h of the injury for mild head injuries. Elevated S100B values are a predictive factor for acute damage on CT scans. Additionally, the Scandinavian guidelines recommend using S100B in CT decision making within 24 h of a head injury. Biochemical determinations are not a permanent element in TBI management regimens. The Brain Trauma Foundation guidelines indicate recommendations for clinical monitoring and therapy (5, 38, 45–47).

Glial fibrillary acid protein (GFAP) is an intermediate filament molecule of the glial cell cytoskeleton, and its molecular weight is 50 kDa. Bolton and Saatman describe GFAP as specific for the central nervous system due to its immunoreactivity, which makes it possible to use the protein as a brain injury biomarker (44, 48). Under normal conditions, the GFAP half-life is 24–48 h, and its plasma levels range from 7 to 20 pg./mL. In cases of TBI, GFAP peaks within 20–24 h, oscillates from 69 to 1,196 pg./mL, and remains at high levels from 3 to 34 h following injury (42, 49). Clinical studies have shown a correlation between elevated GFAP levels, computed tomography scans, and TBI severity, which has been clinically useful for distinguishing the dispersion of intracranial lesions (21, 50, 51). Abnormal serum GFAP concentrations persist for days after brain injury; thus, GFAP has been presented as a good biomarker for long-term prognoses. The optimal cut-off point for GFAP of 626 pg./mL can help predict severe stages of brain damage, while a level of 22 pg./mL provides confirmation of moderate brain damage. Laboratory analyses are based on ELISAs and lanthanide (LDT) fluorescence immunoassays (11, 38, 45–47, 52).

### 3.2 Biomarkers of neuronal damage

Neuron-specific enolase (NSE), a glycolytic enzyme located in the neuronal cytoplasm, is a better-known neuronal injury marker (53). NSE was identified by Moore and McGregor in 1965 as an isoform composition (αα, ββ, γγ, αβ, and αγ), and it is directly connected with the blood–brain barrier and neurons, while only γγ is typical for central and peripheral neurons. Under normal conditions, NSE is limited to intraneuronal space and is not detected in extracellular space. Hemolysis, hemorrhagic shock, and renal failure decrease the specificity of NSE in TBI diagnoses. The positive features of this biomarker—its high specificity for brain tissue, the dynamics of the serum concentration, and the independence of gender and age—indicate that, in many situations, the clinical course is related to the S100B concentration. One limitation of the use of this marker is its relatively long half-life—over 20 h—which reduces its use in the assessment of brain injury dynamics.

Serum NSE levels above 9 μg/L for adults and above 15 μg/L for children within 24 h after the injury are correlated with mild brain trauma on CT scans (21, 53–55). The isoform αγ is minimally detected in the peripheral tissues, such as in the rectum, bladder, and uterus. In addition, biochemical methods such as chromatography and electrophoresis are used to isolate the molecular forms. Other methods used are radioimmunoassays (RIAs) and enzyme-linked immunosorbent assays (ELISAs) based on antigen–antibody interactions. The ELISA method is the most popular and indicates the total NSE concentration. The main limitation of NSE measurements when using the ELISA method is the deceptively elevated NSE concentrations that occur due to hemolysis. This effect occurs when the cell-free hemoglobin concentration is >0.338 g/L. Nevertheless, today, NSE is presented as a good marker in the diagnosis of severe TBI cases, acute intracranial pathologies, and short-term mortality (11, 38, 46, 47, 52, 56). CSF samples should be stored at −80°C for a maximum of 6 months, and serum samples should be stored for a maximum of 9 months.

Ubiquitin carboxyl-terminal hydrolase L1 (UCH-L1) is a neuronal cytoplasm protein that accounts for 1–2% of all brain-soluble proteins (57). UCH-L1 actively participates in the addition or removal of ubiquitin in metabolized proteins and thus plays a significant role in removing excess amounts of oxygenated or abnormally structured proteins from neurons (58, 59). Under normal conditions, the UCH-L1 molecular weight is 24 kDa (60), and the cerebrospinal fluid UCH-L1 levels are 0.7–15.9 ng/mL on average, whereas these values may range from 44.2 to 218.4 ng/mL in TBI cases (27). Zhang et al. (61) describe UCH-L1 as a feasible biomarker for late complications in severe TBI cases.

### 3.3 Biomarkers of demyelination and axonal damage

The tau protein and neurofilament light polypeptide (NF-L) are elements of the cytoskeleton in nervous cells that are linked to the microtubules and are responsible for stabilizing and binding with neurofilaments and cell organelles; additionally, this is the condition for the distance between the microtubules, which determines the axonal diameter (36).

Tau protein evaluation assays are clinically useful for determining long-term axonal damage in gray matter neurons. Additionally, researchers have reported positive and negative predictive values for this protein, sensitivity and specificity for brain complications such as nasal leakage, and an important role in predicting the incidence of loss of consciousness. The total tau concentration normalizes over 8–12 weeks (11, 52, 62).

NF-L assays are a well-known biomarker of myelinated subcortical white matter axon disruption. The quantification techniques are based on immunoassays. The first ELISA test dedicated to NF-L was developed in 1996 by Rosengren. More sensitive methods such as chemiluminescence immunoassays (CLIAs), electrochemiluminescence (ECL) assays, or single-molecule arrays have been developed and provide better specificity and sensitivity, as well as extremely low detection concentrations. All these methods are carried out with sera, plasma, and CSF measurements. The ELISA method provides assay ranges of 0.5–40 pg./mL in plasma/serum. However, regarding a CSF level of 39–40,000 pg./mL, the limit of detection is 33 pg./mL in CSF and 0.4 pg./mL in plasma/serum. Electrochemiluminescence assays and chemiluminescence immunoassays involve carrying out plasma/serum measurements, with assay ranges of 1–50,000 pg./mL and a limit of detection range of 1.49–5.5 pg./mL. A microfluid platform (ELLA) provides assay ranges of 2.7–10,290 pg./mL, with a limit of detection of 2.7 pg./ mL. The lowest limits of detection (0.038 pg./mL) and quantification (0.174 pg./mL) are described in single molecule array (SIMOA) assays, with ranges of 0.5–500 pg./mL (63). NF-L measurements at admission can be used to discriminate between survivors and non-survivors. Importantly, the initial NF-L levels predict poor 12-month clinical outcomes. The magnitudes of the neurofilament light chain increases in patients with post-traumatic DoC range from 2.4- to 60.5-fold the normal upper limit in cerebrospinal fluid in the 1–3-month and 6-month periods after brain trauma, respectively. Additional data suggest that serum NF-L and S100B assays may be useful for predicting long-term neurological outcomes after brain injury. Moreover, the NF-L levels do not differ between patients with hemorrhagic and non-hemorrhagic TBI (63, 64).

Neurofilament (NF) proteins are CNS-specific intermediate filament proteins that are found in neuronal axons and dendrites, and they are 10 nm in diameter (65). NF proteins are composed of polypeptide subunits of various molecular weights, and they assume the form of light chains (an NF-L level of 68 kDa), medium chains (an NF-M level of 145–160 kDa), or heavy chains (an NF-H level of 200–220 kDa) (63). Under normal conditions, serum NF-L levels range from 11 to 17 pg./mL, and values from 89 to 413 pg./mL indicate axonal injury (58, 63, 66, 67). The NFL level in CSF is described as a main sensitive-fluid biomarker of axonal brain injury (23).

The tau protein is mainly expressed in thin, nonmyelinated axons of cortical interneurons, whereas the NFL level is an element in the large-caliber myelinated axons in the deep brain structures and spinal cord. As a result of proteolysis, a cleaved tau protein of 17 kDa is selected. Under normal conditions, the serum tau protein levels fall within 4.48–66.54 pg./mL. These levels increase to 36.44–192.34 pg./mL in patients diagnosed with traumatic brain injury and then normalize 8–12 weeks after the trauma (23, 68–70). The greater amplitude of the changes in the NFL concentration compared to those of the tau concentration indicates that mild TBI is associated with damage to the long myelinated axonal fibers in gray matter and not with the short unmyelinated axonal fibers in the cerebral cortex (23). In one study, the analytical sensitivity for NF-L levels was lowest for SIMOA, higher for ECL and highest for ELISA. Correlations between the paired CSF and serum samples were the strongest for the SIMOA assay (*r* = 0.88, *p* < 0.001) and the ECL assay (*r* = 0.78, *p* < 0.001), while the correlation was weaker in the ELISA measurements (*r* = 0.38, *p* = 0.030). The NF-L levels in the cerebrospinal fluid measurements between the platforms were highly correlated (*r* = 1.0, *p* < 0.001), as well as the serum NF-L levels of the ECL and SIMOA assays (*r* = 0.86, *p* < 0.001); however, the correlations were weaker between the ELISA-ECL assay (*r* = 0.41, *p* = 0.018) and ELISA-SIMOA (*r* = 0.43, *p* = 0.013) (71).

The myelin basic protein (MBP) is an ingredient of CNS oligodendrocytes and a key structural component of multilayer myelin sheath-covering nerve fibers, which play the role of an insulator that accelerates the axonal impulse conduction velocities (72). Demyelination changes lead to the degradation of axons and the myelin sheath, which results in the permeation of the MBP and its fragment into the cerebrospinal fluid or blood (73). The serum BMP levels peak at 48–72 h after subacute traumatic brain injury (74–76). Amyloid precursor protein (APP) accumulates in neurons and axons after brain injury and causes secondary axonal damage. An experimental TBI indicates APP accumulation after 2–3 h (23).

## 4 Systemic markers

The cytokine group is characterized by a complex pleiotropic mechanism of the induction and regulation of local and systemic inflammation. Cytokines are produced locally in elements of brain tissue, as well as via peripheral immune cells, which disturb the assessment of local processes. Immediate gene expression, a rapid increase in the cytokine concentration in body fluids and brain tissue, and a short half-life indicate the favorable properties of these substances as biomarkers. However, their systemic origin and impact are emphasized by their low specificity in relation to brain tissue (21). Pro-inflammatory interleukins augment adverse changes and cell apoptosis, thereby increasing apoptosis protein transcription and intensifying oxidative stress (37, 77). IL-6 and IL-8 determinations are the most widely used, and they reach their peak levels in brain cells on day one after injury and are then considerably elevated on days three to five following the stimulus (78). Under normal conditions, the serum levels are at most 1.8 pg./mL for IL-6 and at most 14.6 pg./mL for IL-8. In TBI cases, the levels are 1,100 pg./mL and 0–2,400 pg./mL, respectively (79, 80). IL-10 is important in the pathogenesis of post-traumatic changes and the regeneration of nervous brain tissue. Local anti-inflammatory action seals the blood–brain barrier and promotes the reconstruction and myelination of nerve fibers, neurons, and astrocytes. The involvement of IL-10 in neuroprotection, neurogenesis, and the regulation of the stress response and hippocampal synaptic plasticity connected with learning and memory has been suggested. Moreover, markers of oxidative stress and antioxidative capacity have been presented as crucial in the pathomechanism of brain tissue injury. The studies demonstrate the association of increased values of specific interleukins (e.g., IL-6, IL-8, IL-10) with increased protein indices (S100B, NSE, GFAP) specific to nervous tissue. Conversely, the literature on cortisol and other specific TBI biomarkers is relatively limited (81–85).

A similar application in TBI prognoses has been described for cortisol measurements. The dynamics of the changes in the cortisol concentrations in blood serum results from the low specificity of this substance toward nervous tissue. Concentration disturbances reflect the hormonal state of the hypothalamic–pituitary–adrenal axis and are also a factor of systemic stress. The description of the bioavailability is noteworthy—determinations in saliva and 24-h urine collections have diagnostic value comparable to that of determinations in blood serum. Tumor necrosis factor (TNF)-alpha assays have little clinical significance due to their systemic origin and effects. In addition, coagulation tests [i.e., the prothrombin time (PT)/International Normalized Ratio (INR)] and the platelet count in peripheral blood counts show that the hemostasis state is not a specific enough biomarker in relation to TBI. The general usefulness of these indices for predicting systemic prothrombotic complications or progressive hemorrhagic changes has been indicated (21). The basic characteristics of the clinical utility of the specific and non-specific biomarkers are presented in Tables 1, 2.

**Table 1:** Basic data and clinical utility of the specific neuronal biomarkers.

Biomarker	Injury information	Normal value	Critical value in TBI	Clinical utility	
S100B	Astrocyte damage	0.06–0.13 μg/L (serum)	Mild TBI: 0.07–0.24 μg/L (serum)	Mild TBI: Sensitivity of 95% Specificity of 29%	Severe TBI: Sensitivity of 61% Specificity of 69%, (38, 46, 47)
GFAP	Astrocyte damage	7–20 pg./mL (plasma)	Mild TBI: 69–1,196 pg./mL (serum)	Moderate TBI: Sensitivity of 93% Specificity of 36%	Severe TBI: Sensitivity of 71% Specificity of 71%. (11, 46, 47, 52)
NSE	Neuronal damage	≤0.15 μg/L (serum)	> 9 μg/L (adult) >15 μg/L (children) (serum)	Severe TBI: Sensitivity of 79% Specificity of 50%.	Mild TBI: Sensitivity of 72% Specificity of 66% (21, 53–55)
UCH-L1	Neuronal damage	0.7–15.9 ng/mL (cerebrospinal fluid)	44.2–216.4 ng/mL (cerebrospinal fluid)	Sensitivity of 97% Specificity of 40%	(27, 63)
NF-L	Axonal white matter damage	11–17 pg./mL (serum)	Severe TBI: 89–413 pg./mL (serum)	Sensitivity of 71% Specificity of 88%.	(58, 63, 66, 67)
Tau	Axonal gray matter damage	2.48–66.54 pg./mL (serum)	Severe TBI: 36.44–192.34 pg./mL (serum)	Sensitivity of 92% Specificity of 100%	(23, 68–70)

**Table 2:** Basic data and clinical utility of the non-specific neuronal biomarkers.

Biomarker	Injury information	Normal value	Critical value in TBI	References
IL-6	Inflammation	≤1.8 pg./mL (serum)	Severe TBI: 0–1,100 pg./mL in serum	Crichton et al. (79) and Kosciuczuk et al. (81)
IL-8	Inflammation	≤14.6 pg./mL (serum)	Severe TBI: 0–2,400 pg./mL (serum)	Crichton et al. (79) and Kaminska et al. (80)
IL-10	Inflammation	4.8–9.8 pg./mL	< 10 pg./mL (serum)	Niiranen et al. (82) and Krausz et al. (84)

## 5 Future of TBI biomarkers

Non-coding, single-stranded RNA molecules with a length of 21–23 nucleotides act as post-transcriptional regulators of gene expression and are involved in mRNA degradation and repression. MicroRNAs, non-coding, single-stranded RNA molecules, have been expressed in the cerebellum, hippocampus, midbrain, and frontal cortex (86) and play a significant role in synapse formation, protein expression, and neuronal network construction (86–88). The significant diagnostic and prognostic value of miRNAs has been described in blood serum and cerebrospinal fluid for MiR-16, MiR92a, and MiR-765 (89, 90). Other miRNA molecules (miR-142-3p, miR-423-3p, miR-425-5p) enable the identification of patients with mild TBI who are at risk of post-brain trauma syndrome and indicate the significant diagnostic and prognostic role of neurotrauma within 48–72 h of the injury (91, 92).

Moreover, the evaluation of salivary miRNAs has shown potential in TBI diagnosis and prognosis assessment. The salivary miRNAs (miR-182-5p, miR-221-3p, mir-26b-5p, miR-320c, miR-29c-3p, and miR-30e-5p) indicate a functional relationship with neuronal development and describe persistent dysregulation for up to 2 weeks after injury (88, 93). miR-27a-5p regulates the sensitivity of neurons to apoptosis and is significant for the protection of the blood–brain barrier (94, 95). The miR-320c values change in both the blood and saliva in severe and mild traumatic brain injury, and the cerebrospinal fluid concentration corresponds with the cerebral cortex (96). Compared to protein markers of traumatic brain injury, miRNAs achieve higher sensitivity due to their stability in peripheral fluids, their ability to cross the blood–brain barrier, and their protection via RNA-binding proteins and exosomes (10, 97).

Biomarker determination in CSF and brain tissue certainly has the greatest medical value. The microdialysis method requires the installation of a special sensor in the brain tissue, as well as other additional bedside devices that enable the reading of the parameters. Unfortunately, this is not an available diagnostic method in every situation. For this reason, the determination of biomarkers in other body fluids is of considerable scientific interest (21).

Cerebrospinal fluid forms the natural brain space and is highly recommended for determining the presence of biomarkers that are initially released from the brain tissue [i.e., S100B, GFAP, UCH-L1, and neurofilaments (NFLs)]. The CSF is an indicator of the tightness of the blood–brain barrier (BBB), as BBB damage is indicated when the serum albumin ratio and the CSF/serum ratio for albumin are increased (10, 23). Because the BBB is disturbed and different mediators (cytokines, cortisol, and TNF) passively penetrate into the CSF and reach higher concentrations than those in the serum, it cannot be understood as a completely true indication of the local intensity of inflammatory processes (23).

Due to the indicated limitations, studies have evaluated the biomarker concentrations in other biological fluids and tissues, such as in whole blood, brain tissue, saliva, urine, and gastric mucosa and via bronchial lavage and oral swabs (21, 24). Most biomarkers in blood have incredibly trace concentrations compared to those in CSF due to the larger distribution volume, the larger intravascular and extracellular water volume, proteolytic degradation, hepatic or renal metabolism, and elimination. For these reasons, there are limitations to the use of blood/serum determinations (23). A small number of experimental studies have presented biomarkers in other body fluids, such as saliva and urine, as well as in fatal cases, which are based on autopsy tests (25, 98). This aspect is the least known and is a space for enriching our knowledge (17, 25, 98–103).

Saliva and urine analyses of patients with TBI may be a real source of molecular biomarkers (104, 105). Janigro et al. (106) presented the clinical utility of assessing the S100B protein levels from the saliva of patients with TBI. In their analysis, the S100B levels were practically four times higher in the saliva samples than those in the control blood samples of patients with suspected TBI. Monroe et al. (107) described a direct correlation between the values of the filament protein light chains (NF-L) obtained from saliva samples and the risk of axonal damage among athletes. Moreover, based on an analysis of saliva samples, Hicks et al. (87) demonstrated that six miRNAs significantly change in patients with traumatic brain injury and thus suggest their use as non-invasive biomarkers of TBI. Through an analysis of the urine samples of patients with traumatic brain injury, Rodriguez et al. (108) showed differences in the S100B kinetic patterns when compared to the blood results. In both analyzed cases, the peak values were reached within 6 h of the injury, and the S100B concentration in the urine decreased gradually by 48 h (but lasted 96 h in the serum) (109). The tau protein has been detected in urine in post-mortem examinations, indicating that this result is a predictive factor of axonal injury, which may be an auxiliary tool for future research and the diagnoses of TBI cases (110–113).

The details of the methodologies and significant technological development have increased the sensitivity and specificity of tests. The mainstream assays used to quantify biomarker levels are enzyme-linked immunosorbent assays (ELISA), polymerase chain reactions (PCRs), mass spectrometry, chromatography, and electrochemical methods. Nevertheless, these methods do not provide the accurate detection of biomarkers at low concentrations. To address this challenge, ultrasensitive methods have been developed, such as digital PCR, rolling-circle amplification (RCA) for the detection of nucleic acids, and meso-scale discovery (MSD) based on electrochemiluminescence technology (114). In 2010, Walt et al. (113, 114) developed a highly sensitive array sensing technology called the single-molecule array (SIMOA). Based on high-density, fiber-optic arrays, the SIMOA allows for the determination of molecules at the single-molecule level, making it a pivotal tool for single-molecule research. Conventional immunoassays, including enzyme-linked immunosorbent assays (ELISAs), chemiluminescence assays, and electrochemiluminescence assays, present low sensitivity levels of approximately 10–13 M (~0.1 pM), while plasma mass spectrometry limits the assay accuracy and is unable to provide quantitative responses. A more widely used technique is immuno-PCR, which increases the sensitivity via the labeling of a detection antibody with a DNA molecule, which is then amplified and quantified via PCR. The sensitivity of these tests has increased 10- to 100-fold over that of conventional immunoassays. An advance in automated immunoassays is the SIMOA analyzer, which integrates the single-molecule sensitivity and multiplexing capability with high-throughput ELISA reagent automation to create an instrumental system capable of molecular-level analysis.

The average sensitivity improvement in SIMOA immunoassays versus conventional ELISAs is more than 1,200-fold, with coefficients of variation of <10% with the limit of detection in fg/mL. The technical challenges of detecting microRNAs in biological fluids are related to the availability of specialized laboratory equipment and appropriately qualified laboratory and medical personnel to perform the sampling. In addition, research on miRNA expression and its diagnostic and prognostic value in TBI is still under scientific evaluation and does not represent a defined standard for evaluation and interpretation. In clinical practice, these factors are the fundamental basis for microRNA analysis (71, 114).

## 6 Conclusion

**Figure 1:**
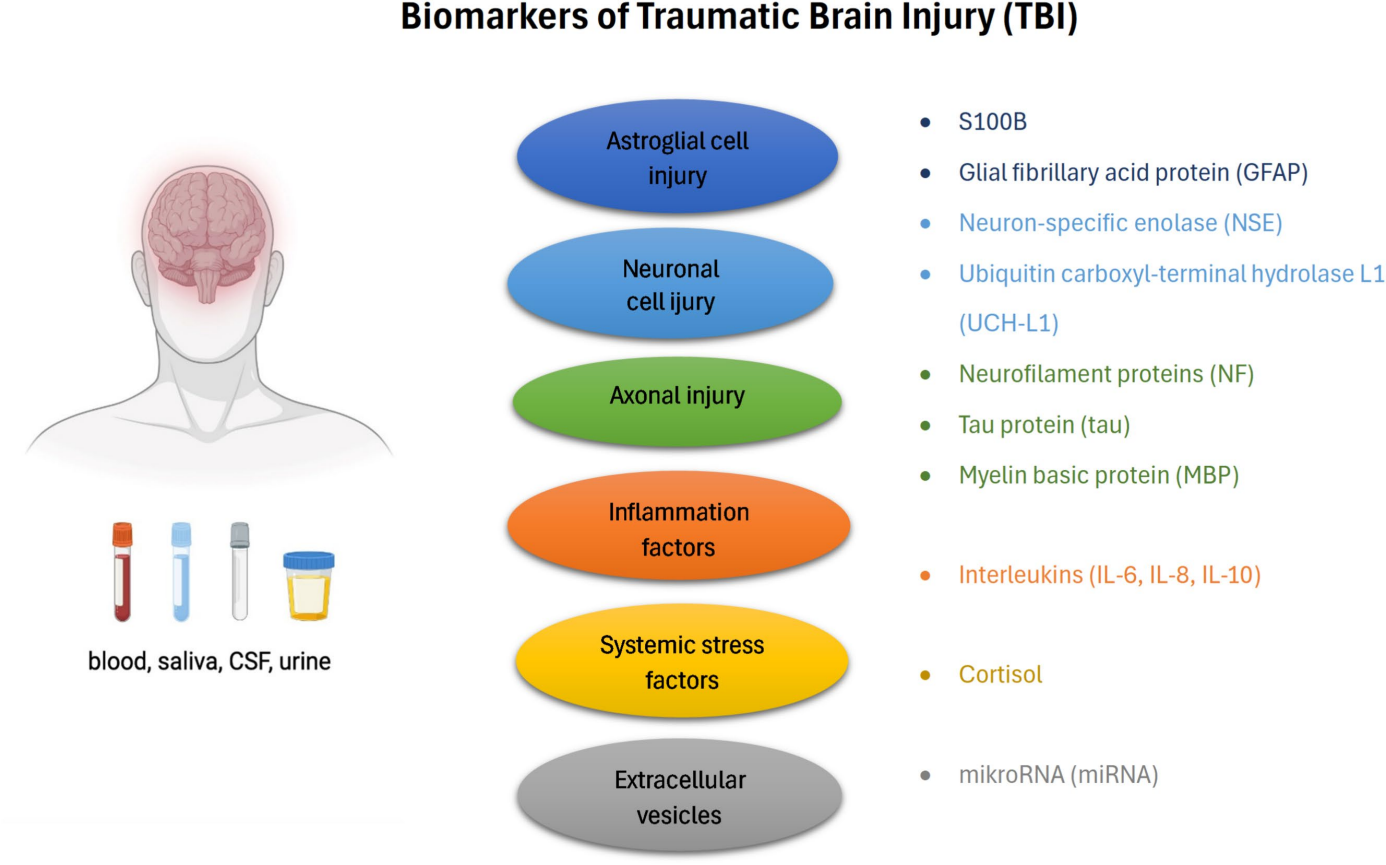
The basic classification of TBI biomarkers (according to the damage and place of origin) and biofluids.

Complex diagnosis schemes based on radiological scans and clinical observations are the main methods for predicting the risk factors of mortality and the neurological state. Various biomarkers have been presented as acute- and chronic-phase TBI mediators that indicate the degree of the brain injury and the neurological condition in critical care (Figure 1). The crucial advantages and clinical prognosis basic on TBI biomarkers are presented in Table 3.

**Table 3:** Crucial advantages and clinical prognosis basic on TBI biomarkers.

Specific neuronal biomarker	Crucial advantages	Clinical prognosis
Biomarkers of astroglial cell damage S 100B	Relatively unaffected by external environment conditions, hemolysis, storage conditions.	Diagnosis of acute morphological brain pathologies recommended using in decision making within 24 h of head injury.
Biomarkers of astroglial cell damage GFAP	Half-life is 24–48 h.	Abnormal concentrations persist for days afetr brain injury Good biomarker of long term neurological prognoses.
Biomarkers of neuronal damage NSE	High specificity for brain tissue. Half-life is 20 h.	Diagnosis of acute intracranial pathologies and short term mortality. Long term neurological prognosis.
Biomarkers of demyelination and axonal damage Tau and NF -L	Tau- gray matter injury. NF-L white matter injury.	Long term neurological prognosis (over 8–12 weeks).

Based on the available publications, it can be stated that there is no definitive and accurate single marker with a high prognostic value for neurological damage to brain tissue; however, the combination of several substances significantly increases the diagnostic value. This approach allows for a holistic assessment of the brain injury stages and for predictions of any complications. Serum and CSF biomarkers are a promising prognostic and diagnostic tool for TBI. There are no additional regulations on the collection and use of other biological materials for diagnostics. Future trends in studies on the markers of brain tissue damage are concentrated on the creation of the optimal brain injury biomarker panel that considers genetic analyses, specific mRNAs, and the use of other biofluids in laboratory examinations when using ultra-sensitive technology, which will allow for the application of the appropriate therapies, thereby reducing the number of complications and the risk of death in patients diagnosed with traumatic brain injury.
